# Virucidal Activities of Acidic Electrolyzed Water Solutions with Different pH Values against Multiple Strains of SARS-CoV-2

**DOI:** 10.1128/aem.01699-22

**Published:** 2022-12-13

**Authors:** Yohei Takeda, Masaru Nikaido, Dulamjav Jamsransuren, Sachiko Matsuda, Haruko Ogawa

**Affiliations:** a Research Center for Global Agromedicine, Obihiro University of Agriculture and Veterinary Medicine, Obihiro, Hokkaido, Japan; b Department of Veterinary Medicine, Obihiro University of Agriculture and Veterinary Medicine, Obihiro, Hokkaido, Japan; c Food Ingredients Department, Morinaga Milk Industry Co., Ltd., Tokyo, Japan; University of Nebraska-Lincoln

**Keywords:** acidic electrolyzed water, disinfectant, pH, SARS-CoV-2, variant strain

## Abstract

Severe acute respiratory syndrome coronavirus 2 (SARS-CoV-2) is a threat to human health. Acidic electrolyzed water (AEW) has recently been suggested to demonstrate virucidal activity. Many types of AEW with different pH values, generated by the electrolysis of different chemicals, such as sodium chloride, potassium chloride, and hydrochloric acid, are commercially available. In this study, we compared the virucidal activities of these types of AEW against SARS-CoV-2, including the ancestral strain and variant Alpha, Beta, Gamma, Delta, and Omicron strains. Virus solution (viral titer, 6.9 log_10_ 50% tissue culture infective dose [TCID_50_]/mL) was mixed with AEW (free available chlorine concentration, 34.5 ppm) at mixing ratios of 1:9, 1:19, and 1:49. At mixing ratios of 1:9 and 1:19, AEW with a pH of 2.8 showed stronger virucidal activities than AEW with a pH of 4.1 to 6.5 against the SARS-CoV-2 ancestral strain in 20 s. From the strongest to the weakest virucidal activity, the AEW pH levels were as follows: pH 2.8, pH 4.1 to 5.4, pH 6.4 to 6.5. At a ratio of 1:49, the viral titers of viruses treated with all AEW solutions at pH 2.8 to 6.5 were almost below the detection limit, which was 1.25 log_10_ TCID_50_/mL. The virus inactivation efficiency of AEW was reduced in the presence of fetal bovine serum and other substances contained in the virus solution used in this study. AEW with pH values of 2.8 to 6.5 showed virucidal activity against all of the tested SARS-CoV-2 strains, including the ancestral and variant strains. These results provide useful knowledge for the effective application of AEW as a SARS-CoV-2 disinfectant.

**IMPORTANCE** Acidic electrolyzed water (AEW) demonstrates virucidal activity against multiple viruses. Since AEW exhibits low toxicity, is inexpensive, and is environmentally friendly, it can be a useful disinfectant against severe acute respiratory syndrome coronavirus 2 (SARS-CoV-2). Although the pH values of currently available AEW products vary, the impact of different pH values on SARS-CoV-2 inactivation has not previously been evaluated in detail. In this study, we compared the virucidal activities of multiple AEW solutions with different pH values, under the same experimental conditions. We found that AEW solutions with lower pH values demonstrated more potent virucidal activity. Also, we showed that the extent of virus inactivation by the AEW was based on the balance of the abundance of free available chlorine, virus, and other organic substances in the mixture. AEW exhibited rapid virucidal activity against multiple SARS-CoV-2 strains. This study demonstrated the usefulness of AEW as a disinfectant which can be applied to the inactivation of SARS-CoV-2.

## INTRODUCTION

The coronavirus disease 2019 (COVID-19) pandemic caused by severe acute respiratory syndrome coronavirus 2 (SARS-CoV-2) infection is still ongoing as of September 2022. The ongoing emergence of variant strains with altered antigenicity, transmissibility, and phenotypic characters has prolonged the pandemic situation (1). Surges in COVID-19 cases have repeatedly overwhelmed and induced the collapse of medical systems, and infection prevention measures implemented by individuals play important roles in the control of SARS-CoV-2. Current vaccines induce host immune responses against variant strains with altered antigenicity, including the Omicron strain, with repeated vaccination (1, 2). However, the rate of change of antigenicity in the variants exceeds that of new vaccine development, and a real risk exists of the emergence of new variants which can escape the currently established immunity. In such a situation, hand hygiene, mask wearing, social distancing, and ventilation are regarded as important SARS-CoV-2 infection protective measures (3, 4), along with vaccination. In addition to transmission of SARS-CoV-2 by inhalation of respiratory droplets/aerosols, direct SARS-CoV-2 transmission via contaminated hands may possibly occur (5) (https://www.cdc.gov/coronavirus/2019-ncov/science/science-briefs/sars-cov-2-transmission.html). However, indirect transmission from contaminated environmental surfaces is considered to occur only rarely (6) (https://www.cdc.gov/coronavirus/2019-ncov/science/science-briefs/sars-cov-2-transmission.html). Nevertheless, infectious SARS-CoV-2 has been detected not only on COVID-19 patients’ hands but also on environmental surfaces (7). Therefore, many medical sectors have incorporated not only hand hygiene but also cleaning of environmental surfaces into their guidelines for the strict control of SARS-CoV-2 infection.

Acidic electrolyzed water (AEW), also called hypochlorous acid water, is water generated by electrolyzing dilute sodium chloride (NaCl), potassium chloride, or hydrochloric acid (HCl) in an electrolysis chamber. AEW generated in electrolysis chambers with one, two, or three compartments is clearly distinguishable from solutions created by mixing sodium hypochlorite and acidic solutions. Because of their low toxicity, cost, and environmental impact, AEW solutions have recently become widely used as chlorine-based disinfectants for various types of pathogenic microorganisms, including bacteria, fungi, and viruses (8, 9). In Japan, where studies of AEW have been conducted since around 1930, some types of AEW are used as food additives for the disinfection of pathogens on the surface of foods, as designated by Ministry of Health, Labor, and Welfare in 2002 (9), and as inactivating agents for crops, according to the Ministry of Agriculture, Forestry, and Fisheries in 2014. Organizations in multiple countries, including the U.S. Food and Drug Administration, have authorized the use of AEW as a safe disinfectant for food, human skin, and environmental surfaces (9).

AEW primarily contains three different forms of chlorine: HClO, ClO^−^, and Cl_2_. The ratio of these chlorines changes depending on the pH of the AEW (10). The germicidal activity of HClO is stronger than that of ClO^−^ (9). The differences in the types of chemical compounds, such as NaCl or HCl, used for AEW generation and the different types of electrolysis chambers—one, two, or three compartments—seem to be factors influencing the character of AEW, like the stability of free available chlorine (FAC) in the solution. Although many types of commercially available AEW are found, with different pH values, few studies exist comparing the activities of these different AEW solutions under the same experimental conditions. AEW has been reported to demonstrate virucidal activity against a wide range of pathogenic viruses, including influenza A virus (11, 12), African swine fever virus (12), foot-and-mouth disease virus (13), porcine reproductive and respiratory syndrome virus and pseudorabies virus (14), hepadnavirus (15), human immunodeficiency virus (16), norovirus (17), herpes simplex viruses, and more (18). We previously reported that some types of AEW inactivated the ancestral strain (lineage A) of SARS-CoV-2 that first emerged in 2019 and was isolated in the early stages of the COVID-19 pandemic (19, 20). In the current study, we compared the virucidal activities of several types of AEW with different pH values, which were generated by the electrolysis of different chemicals, against multiple SARS-CoV-2 strains, including variants, to clarify the effectiveness of each AEW for use as a SARS-CoV-2 disinfectant.

## RESULTS

### Virucidal activities of AEW against the ancestral strain of SARS-CoV-2.

**FIG 1:**
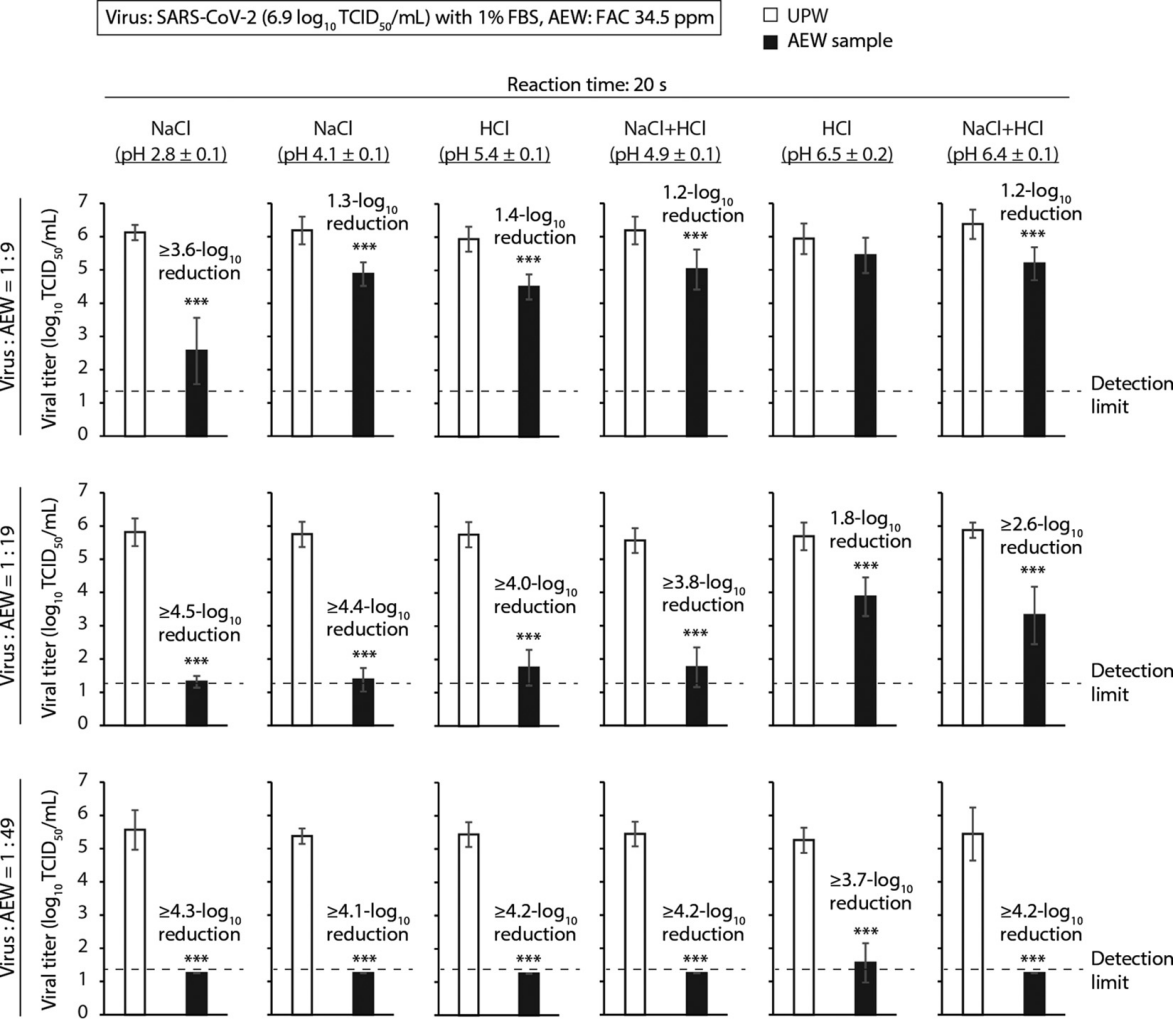
Virucidal activity of various types of AEW against the SARS-CoV-2 ancestral strain. VGM containing SARS-CoV-2 (ancestral strain) with 1% FBS (viral titer, 6.9 log_10_ TCID_50_/mL) was mixed with multiple AEW solutions (FAC concentration, 34.5 ± 1.9 ppm) at ratios of 1:9 (top), 1:19 (middle), and 1:49 (bottom). As a control, the virus solution was mixed with ultrapure water (UPW). After 20 s, the viral titer of each mixture was evaluated. The detection limit of the viral titer was 1.25 log_10_ TCID_50_/mL. Error bars indicate mean ± SD (*n* = 8). Student’s *t* tests were performed to analyze the statistical significance of the differences between the UPW and each AEW group; ***, *P < *0.001.

The virucidal activities of six AEW treatments against the SARS-CoV-2 ancestral strain were evaluated. These treatments were generated by electrolyzing NaCl solution, HCl solution, or a mixture of NaCl and HCl solutions. These different AEW treatments had different pH values. Based on the chemicals used for electrolysis and the pH of the solution, the six tested AEW solutions in this study are referred to as NaCl (pH 2.8 ± 0.1), NaCl (pH 4.1 ± 0.1), HCl (pH 5.4 ± 0.1), NaCl+HCl (pH 4.9 ± 0.1), HCl (pH 6.5 ± 0.2), and NaCl+HCl (pH 6.4 ± 0.1). In Japan, the recommended FAC concentration of AEW for SARS-CoV-2 disinfection has been announced as 35 ppm or higher, based on a study led by the National Institute of Technology and Evaluation (https://www.nite.go.jp/data/000115863.pdf). However, there is also a demand to acquire knowledge about the SARS-CoV-2-inactivating activity of AEW with lower FAC concentrations, which may result in less skin irritation. Therefore, the virucidal activities of AEW treatments with two different FAC concentrations, 34.5 ± 1.9 ppm and 23.8 ± 1.6 ppm, were evaluated in this study. First, the treatments with FAC concentrations of 34.5 ± 1.9 ppm were evaluated. When SARS-CoV-2-containing viral growth medium (VGM) with 1% fetal bovine serum (FBS) (viral titer, 6.9 log_10_ 50% tissue culture infective dose [TCID_50_]/mL) and each AEW solution were mixed at a ratio of 1:9 by volume, NaCl AEW (pH 2.8 ± 0.1) caused a ≥3.6-log_10_ TCID_50_/mL reduction in the viral titer in 20 s reaction time; this type of AEW showed the most potent virucidal activity of the six treatments tested. The other five treatments caused no or limited reduction in viral titer within the same period of treatment. HCl AEW (pH 6.5 ± 0.2) did not cause a statistically significant reduction in the viral titer; the other four AEW solutions caused reductions of 1.2 to 1.4 log_10_ TCID_50_/mL (Fig. 1, top). At a mixing ratio of 1:19, NaCl AEW (pH 2.8 ± 0.1) and NaCl AEW (pH 4.1 ± 0.1) showed the first and second strongest virucidal activities, respectively, causing ≥4.5- and ≥4.4-log_10_ TCID_50_/mL reductions, respectively. HCl AEW (pH 5.4 ± 0.1) and NaCl+HCl AEW (pH 4.9 ± 0.1) also produced reductions of ≥4.0 and ≥3.8 log_10_ TCID_50_/mL, respectively. HCl AEW (pH 6.5 ± 0.2) and NaCl+HCl AEW (pH 6.4 ± 0.1) showed reductions of 1.8 and ≥2.6 log_10_ TCID_50_/mL, respectively (Fig. 1, middle). At a mixing ratio of 1:49, the viral titers in all six AEW solutions were almost below the detection limit, with reductions of ≥3.7 to ≥4.3 log_10_ TCID_50_/mL (Fig. 1, bottom). In addition, the virucidal activities of AEW solutions with a lower FAC concentration (23.8 ± 1.6 ppm) were evaluated. When virus-containing VGM with 1% FBS (viral titer, 6.9 log_10_ TCID_50_/mL) and AEW were mixed at a ratio of 1:9, NaCl (pH 2.8 ± 0.1) caused a ≥4.1-log_10_ TCID_50_/mL reduction in 20 s. NaCl (pH 4.1 ± 0.1) showed very weak virucidal activity (a 0.5-log_10_ TCID_50_/mL reduction), and the other four AEW solutions did not show statistically significant virucidal activity (see Fig. S1A in the supplemental material). When virus-containing VGM with 1% FBS with a lower viral titer (4.9 log_10_ TCID_50_/mL) and AEW were mixed at a ratio of 1:9, the viral titers in all six AEW groups were reduced below the detection limit, with reductions of ≥2.5 to ≥3.0 log_10_ TCID_50_/mL (Fig. S1B). These results indicate that NaCl AEW (pH 2.8 ± 0.1) has more potent virucidal activity than other five AEW solutions tested. These results also suggest that the SARS-CoV-2 inactivation efficacy of AEW increases when the FAC concentration is higher, the liquid/volume ratio of AEW to the virus solution is higher, and the viral titer of the virus solution is lower.

To evaluate the impact of acidic pH on the FAC-independent virucidal activity of AEW, AEW solutions with an FAC concentration of 0.0 ppm were tested. When virus-containing VGM with 1% FBS (viral titer, 6.9 log_10_ TCID_50_/mL) and AEW with an FAC concentration of 0.0 ppm were mixed at a ratio of 1:19, NaCl AEW solutions at pH 3.1 ± 0.0, pH 5.5 ± 0.1, and pH 6.4 ± 0.0 showed no statistically significant virucidal activity in 20 s or 3 h (Fig. S2, top, middle). In 24 h, NaCl AEW at a pH of 3.1 ± 0.0 produced a ≥3.6-log_10_ TCID_50_/mL reduction in the viral titer. The other two solutions showed no or very limited virucidal activity (Fig. S2, bottom).

### Change in pH and chlorine concentration in AEW after mixing with VGM.

**FIG 2:**
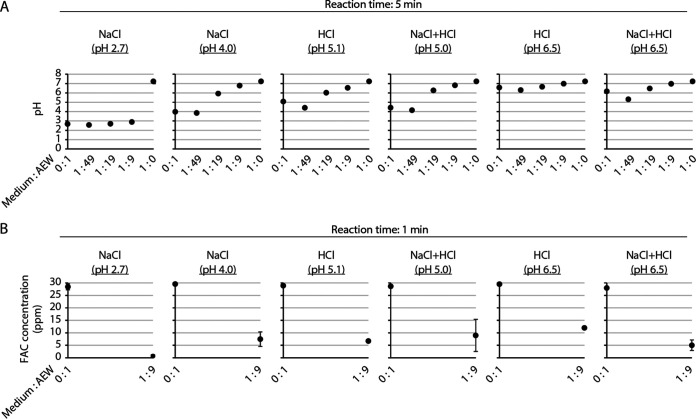
Changes in pH and FAC concentration in AEW groups after mixing with VGM. (A) pH of the mixture of virus-free VGM and each AEW at various mixing ratios was measured after 5 min. (B) FAC concentrations in the mixture of virus-free VGM and each AEW at a ratio of 0:1 or 1:9 were measured after 1 min. Error bars indicate mean ± SD (*n* = 2).

Virus-free VGM with 1% FBS and AEW with an FAC concentration of 30.0 ppm were mixed at various ratios, and the pH values of the mixtures were measured. The pH of VGM without AEW (medium-to-AEW ratio of 1:0) was 7.2. The pH values of all six AEW solutions were slightly lower at a medium-to-AEW ratio of 1:49 than at a ratio of 0:1. The pH of NaCl (pH 2.7) AEW at a ratio of 1:9 was comparable to that at a ratio of 0:1. The pH values of the other five AEW solutions were higher at a ratio of 1:9 than at a ratio of 0:1 (Fig. 2A). Next, the change in FAC concentration in each AEW solution was evaluated after mixing with VGM at a medium-to-AEW ratio of 1:9. The reduction in FAC after mixing with VGM was highest for NaCl AEW (pH 2.7), with the FAC concentration reaching almost 0 ppm (Fig. 2B).

### Impact of organic substances on the virucidal activity of AEW.

**FIG 3:**
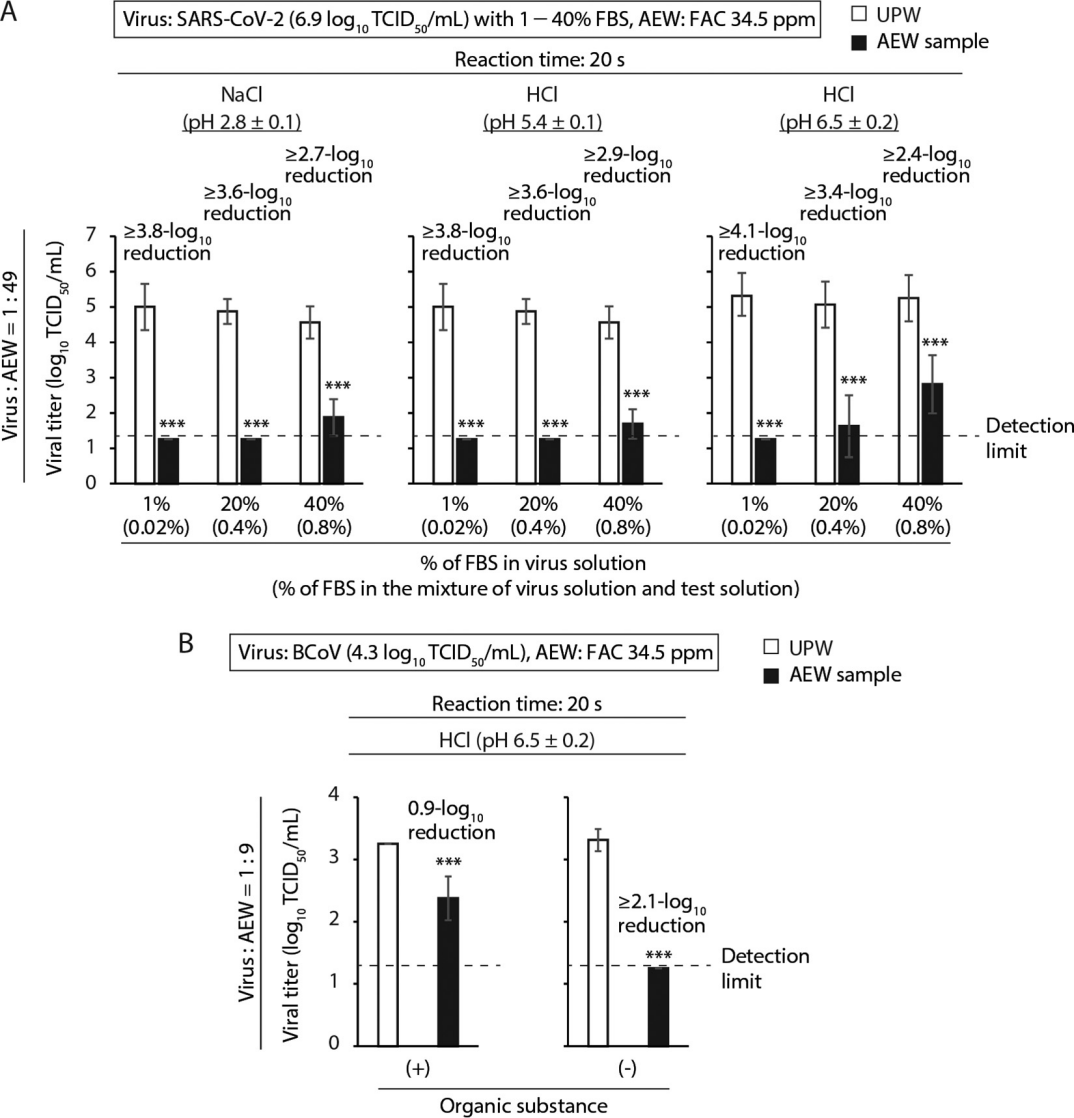
Impact of the presence of organic substances on the virucidal activity of AEW. (A) VGM containing SARS-CoV-2 (ancestral strain) with 1%, 20%, or 40% FBS (viral titer, 6.9 log_10_ TCID_50_/mL) was mixed with multiple AEW solutions (FAC concentration, 34.5 ± 1.9 ppm) at a ratio of 1:49. (B) BCoV-containing VGM with 1% FBS (organic substance [+]) or BCoV-containing PBS (organic substance [−]) (viral titer, 4.3 log_10_ TCID_50_/mL) was mixed with AEW (HCl [pH 6.5 ± 0.2]; FAC concentration, 34.5 ± 1.9 ppm) at a ratio of 1:9. (A, B) As a control, virus solution was mixed with UPW. After 20 s, the viral titer of each mixture was evaluated. The detection limit of the viral titer was 1.25 log_10_ TCID_50_/mL. Error bars indicate mean ± SD (*n* = 8). Student’s *t* tests were performed to analyze the statistical significance of the differences between the UPW and each AEW group; ***, *P* < 0.001.

The virucidal activities of the AEW solutions at an FAC concentration of 34.5 ± 1.9 ppm against SARS-CoV-2 (ancestral strain) in VGM with FBS contents of 1%, 20%, or 40% were evaluated at a virus-to-AEW ratio of 1:49. The virus solution containing a high concentration of FBS mimics viral fluids, with large amounts of organic substances, in the real world. The final concentrations of FBS in the virus and test solution mixture were 0.02%, 0.4%, or 0.8%, respectively. After 20 s, the viral titers decreased to below the limit of detection in the NaCl (pH 2.8 ± 0.1) and HCl (pH 5.4 ± 0.1) groups when the FBS contents in the virus-containing VGM were 1% and 20% FBS, respectively, while these titers were above the detection limit when the FBS content was 40%. In the HCl (pH 6.5 ± 0.2) group, the viral titer was below the detection limit at an FBS content of 1%, while the titer was above the detection limit at FBS contents of 20% or 40%. There was a smaller reduction in the viral titer in the 40% FBS group than in the 20% FBS group (Fig. 3A). To eliminate the influence of multiple compositions of VGM on the virucidal activity of AEW, virus-containing phosphate-buffered saline (PBS) was prepared. SARS-CoV-2-containing PBS could not be prepared due to biosafety restrictions, so bovine coronavirus (BCoV) was used as a surrogate for SARS-CoV-2. BCoV-containing VGM with 1% FBS or BCoV-containing PBS was mixed with HCl AEW (pH 6.5 ± 0.2) at a ratio of 1:9, and the reduction in the viral titer was evaluated. The reduction in the viral titer was very small (0.9 log_10_ TCID_50_/mL) when AEW was mixed with virus-containing VGM, while the reduction was bigger (≥2.1 log_10_ TCID_50_/mL; the viral titer was below the detection limit) when AEW was mixed with virus-containing PBS (Fig. 3B).

### Virucidal activities of AEW against multiple SARS-CoV-2 strains, including variants.

**FIG 4:**
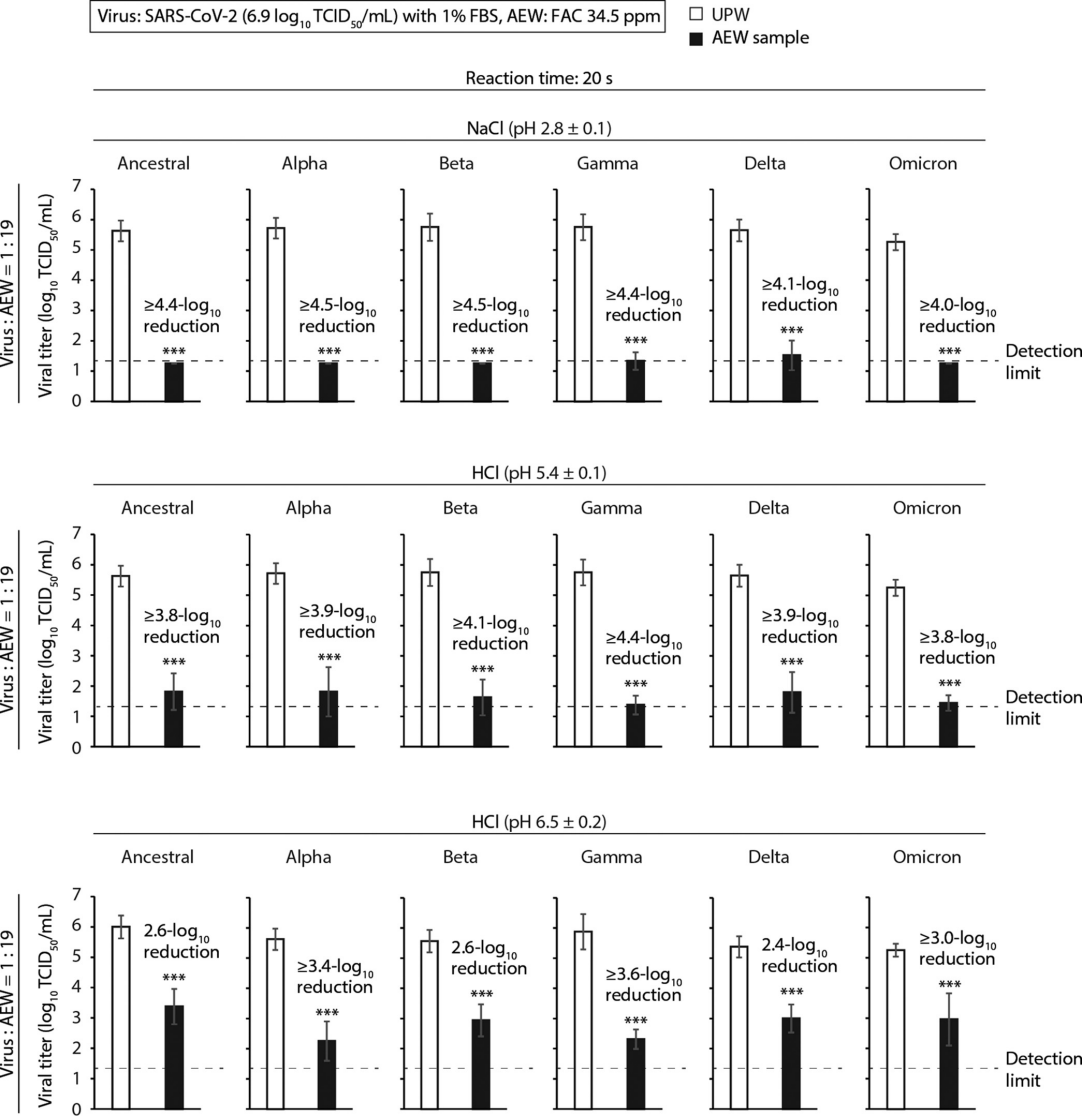
Virucidal activities of AEW against multiple SARS-CoV-2 strains, including variants. VGM containing SARS-CoV-2 (ancestral, Alpha, Beta, Gamma, Delta, and Omicron strains) with 1% FBS (viral titer, 6.9 log_10_ TCID_50_/mL) was mixed with multiple AEW solutions (FAC concentration, 34.5 ± 1.9 ppm) at a ratio of 1:19. As a control, virus solution was mixed with UPW. After 20 s, the viral titer of each mixture was evaluated. The detection limit of the viral titer was 1.25 log_10_ TCID_50_/mL. Error bars indicate mean ± SD (*n *= 8 to 24). Student’s *t* tests were performed to analyze the statistical significance of the differences between the UPW and each AEW group; ***, *P* < 0.001.

The virucidal activities of AEW against SARS-CoV-2 Alpha, Beta, Gamma, Delta, and Omicron variant strains (BA.1 lineage) were compared with those against the ancestral strain. Virus-containing VGM with 1% FBS (viral titer, 6.9 log_10_ TCID_50_/mL) and AEW (FAC, 34.5 ± 1.9 ppm) were mixed at a ratio of 1:19. After 20 s, NaCl AEW (pH 2.8 ± 0.1) and HCl AEW at pH values of 5.4 ± 0.1 and 6.5 ± 0.2 showed comparable or slightly higher virucidal activity against the five different variants as against the ancestral strain. The reductions in viral titer produced by NaCl (pH 2.8 ± 0.1) against the ancestral, Alpha, Beta, Gamma, Delta, and Omicron strains were ≥4.4, ≥4.5, ≥4.5, ≥4.4, ≥4.1, and ≥4.0 log_10_ TCID_50_/mL, respectively. The viral titers of all strains treated with NaCl (pH 2.8 ± 0.1) were almost below the detection limit (Fig. 4, top). The reductions in viral titer produced by HCl AEW (pH 5.4 ± 0.1) against the ancestral, Alpha, Beta, Gamma, Delta, and Omicron strains were ≥3.8, ≥3.9, ≥4.1, ≥4.4, ≥3.9, and ≥3.8 log_10_ TCID_50_/mL, respectively (Fig. 4, middle). The reductions in viral titer produced by HCl AEW (pH 6.5 ± 0.2) against the ancestral, Alpha, Beta, Gamma, Delta, and Omicron strains were 2.6, ≥3.4, 2.6, ≥3.6, 2.4, and ≥3.0 log_10_ TCID_50_/mL, respectively (Fig. 4, bottom).

## DISCUSSION

When virus-containing VGM with 1% FBS (viral titer, 6.9 log_10_ TCID_50_/mL) and AEW solutions (FAC, 34.5 ± 1.9 ppm) were mixed at a ratio of 1:9, NaCl AEW (pH 2.8 ± 0.1) produced more potent virucidal activity against the SARS-CoV-2 ancestral strain than the other five solutions (Fig. 1). At this ratio, the estimated pH of the mixture was 2.9 in the NaCl (pH 2.8 ± 0.1) group and 6.6 to 7.0 in the other five groups (pH 6.8 in the NaCl [pH 4.1 ± 0.1] group, pH 6.6 in the HCl [pH 5.4 ± 0.1] group, pH 6.8 in the NaCl+HCl [pH 4.9 ± 0.1] group, pH 7.0 in the HCl [pH 6.5 ± 0.2] group, and pH 7.0 in the NaCl+HCl [pH 6.4 ± 0.1] group) (Fig. 2A). In the five AEW groups, excluding the NaCl (pH 2.8 ± 0.1) group, the pH appeared to increase, due to the pH-buffering capacity of VGM. Chigusa et al. (21) reported that AEW at a pH of 3.0 showed more potent virucidal activity against feline calicivirus, herpes simplex virus, and influenza A virus than AEW at a pH of 6.0. HClO is in equilibrium with ClO^−^ in aqueous solution, and the pK_a_ value is 7.5 at 25°C. The ratio of HClO and ClO^−^ abundance is determined by the pH. The proportion of ClO^−^ abundance increases as the pH increases, and Cl_2_ is produced at highly acidic pH, in which the equilibrium system between HClO and ClO^−^ is not established (22, 23). In general, the germicidal activity of ClO^−^ appears to be weaker than that of HClO (9), while the strength of the activity of Cl_2_ in a liquid [Cl_2_(aq)] is unknown, because Cl_2_ is immediately released into the air after generation (24). The solubility of Cl_2_ in water was 0.63 g/100 g of H_2_O at 25°C under 0.973 atm (25). Although the change from Cl_2_(aq) to Cl_2_(g) is accelerated under highly acidic conditions, Cl_2_(aq) may exist in such a highly acidic solution, even though the time taken for conversion to Cl_2_(g) is short (26). Cherney et al. (27) argued that Cl_2_(aq) is a powerful oxidant in highly acidic solutions. Since the oxidizing activity of AEW is considered to contribute to the inactivation of microorganisms (9), the strong oxidative activity of Cl_2_(aq) may have also contributed to the potent virucidal activity in highly acidic conditions. Another hypothesis exists regarding the correlation between low pH and potent virucidal activity. The AEW solutions (pH 3.1 to 6.4; FAC, 0.0 ppm) did not show SARS-CoV-2-inactivating activities within 3 h at a ratio of 1:19. AEW solutions at pH 3.1 ± 0.0 (FAC, 0.0 ppm), but not at pH 5.5 ± 0.1 or 6.4 ± 0.0 (FAC, 0.0 ppm), produced potent virucidal activity in 24 h (see Fig. S2 in the supplemental material). This result suggests that highly acidic pH (around 3.0) does not directly contribute to the SARS-CoV-2-inactivating activity of AEW with short reaction times. Nevertheless, *in silico* experiments demonstrated the possibility that highly acidic conditions induce a structural alteration in the spike (S) protein of SARS-CoV-2 (28). Another study showed that the S protein refolded in an acidity-dependent manner, inducing a down conformation of the receptor binding domain (29). The up or down conformation change may be invertible, and the acidic pH itself does not lead to the loss of SARS-CoV-2 infectivity over a short reaction time. However, an acidic pH-dependent conformational change in the S protein might enhance the access of FAC to structures that are critical for the establishment of infection.

When virus-containing VGM with 1% FBS (viral titer, 6.9 log_10_ TCID_50_/mL) and AEW solutions with an FAC concentration of 34.5 ± 1.9 ppm were mixed at a ratio of 1:19, the virucidal activities of the NaCl (pH 4.1 ± 0.1), HCl (pH 5.4 ± 0.1), and NaCl+HCl (pH 4.9 ± 0.1) groups were greater than those of the HCl (pH 6.5 ± 0.2) or NaCl+HCl (pH 6.4 ± 0.1) groups (Fig. 1), although there was no critical difference in the estimated pH of the mixture in these five AEW groups (Fig. 2A). Although the possibility that the very small differences in pH affected the virucidal activity of chlorine in the mixture cannot be completely excluded, the results also suggest the possibility of the presence of factors other than pH that affected the virucidal activity of the AEW.

When the ratio was 1:49, all six AEW groups produced potent virucidal activity (Fig. 1). The absolute amounts of both virus and VGM-derived multiple compositions contained in the mixture were less in the 1:49 mixtures than at ratios of 1:9 and 1:19. On the contrary, the absolute amount of FAC in the mixture was higher at a ratio of 1:49. The strength of the virus inactivation effect seemed to be determined by the balance of the abundances of FAC, virus, and other organic substances in the mixture. Similarly, even when the FAC concentration was low (23.8 ± 1.6 ppm), AEW showed potent virucidal activity, regardless of pH, against SARS-CoV-2 with a low viral titer (4.9 log_10_ TCID_50_/mL) in a 1:9 mixture (Fig. S1). When the absolute amount of substances other than viruses, such as organic substances, was increased, the efficiency of virus inactivation decreased (Fig. 3). This trend was evident in all AEW solutions, regardless of pH. Overall, the differences in chemicals used for AEW generation by electrolysis did not appear to be critical to the disinfectant activity of AEW. One limitation of the current study was that AEW was mixed with virus-containing VGM, which does not exist in the real world. Nevertheless, our findings indicate that the use of large amounts of AEW with a sufficient concentration of FAC, or the removal of organisms before applying AEW, can be used to maximize the disinfectant efficacy of AEW.

The efficacy of inactivation of ethanol, isopropanol, and hand soap containing surfactants against SARS-CoV-2 variant strains has been evaluated. Sufficient concentrations of these disinfectants showed comparable activity against all tested variants (30, 31). In the current study, AEW at pH 2.8 to 6.5 also showed virucidal activity against multiple SARS-CoV-2 variant strains. Among these variants, resistance to the AEW solutions was not observed (Fig. 4). The virucidal mechanisms of action of AEW appear to depend on its high oxidizing activity, which induces disruption of the envelope and genome and the denaturation, aggregation, and destruction of viral proteins (9, 24, 32). Since these functions are nonspecific, the possibility exists that AEW may effectively inactivate emerging strains of SARS-CoV-2.

We showed that AEW at a pH of ≤3.0 demonstrated the strongest SARS-CoV-2-inactivating activity, but AEW at higher pH values also showed good virucidal activity when they had high concentrations of FAC and were used in large volumes. Therefore, AEW at a pH of ≤3.0 appears to be most suitable for the disinfection of highly polluted places, but its short FAC retention period (20) and the risk to users posed by highly acidic pH values should be taken into account. In contrast, the long FAC retention period (20) and low risk of AEW with higher pH values makes it suitable for long-term storage and application to hand hygiene. The corrosive action of AEW, regardless of pH, against some materials, including metals, should also be considered (9, 10, 21). In recent years, the versatility and usefulness of AEW as a disinfectant have become well known. Our findings will provide useful insights into the effective application of AEW to the inactivation of SARS-CoV-2.

## MATERIALS AND METHODS

### Cells and viruses.

Vero E6/TMPRSS2 cells were obtained from the Japanese Collection of Research Bioresources (Osaka, Japan). Multiple SARS-CoV-2 strains (A lineage [ancestral strain], 2019-nCoV/Japan/TY/WK-521/2020 [GISAID identifier ID, EPI_ISL_408667]; lineage B.1.1.7 [Alpha strain], hCoV-19/Japan/QHN001/2020 [GISAID ID, EPI_ISL_804007]; lineage B.1.351 [Beta strain], hCoV-19/Japan/TY8-612-P1/2021 [GISAID ID, EPI_ISL_1123289]; lineage P.1 [Gamma strain], hCoV-19/Japan/TY7-501/2021 [GISAID ID, EPI_ISL_833366]; lineage B.1.617.2 [Delta strain], hCoV-19/Japan/TY11-927-P1/2021 [GISAID ID, EPI_ISL_2158617]; and lineage BA.1 [Omicron strain], hCoV-19/Japan/TY38-873P0/2021 [GISAID ID, EPI_ISL_7418017]) were provided by the National Institute of Infectious Diseases (Tokyo, Japan). Next, SARS-CoV-2-inoculated Vero E6/TMPRSS2 cells were cultivated in VGM consisting of Dulbecco’s modified Eagle’s medium (Nissui Pharmaceutical Co., Ltd., Tokyo, Japan) containing 1% FBS, 2 mM l-glutamine, 2 μg/mL amphotericin B, and 100 μg/mL kanamycin. The pH of the VGM was adjusted to 7.2 by adding NaHCO_3_ solution. HRT-18 Aichi cells and the BCoV strain Mebus were provided by the Aichi Prefectural Chuo Livestock Hygiene Service Center (Okazaki, Japan). BCoV-inoculated HRT-18 Aichi cells were cultivated in VGM. The composition of VGM for the HRT-18 Aichi cells was almost the same as that for the Vero E6/TMPRSS2 cells, but it did not contain amphotericin B. After the virus-inoculated cells were incubated at 37°C for 3 or 4 days, the cell culture supernatants were collected and used as virus-containing VGM. BCoV-containing PBS was prepared as follows: BCoV-containing VGM was ultracentrifuged at 100,000 × *g* for 3 h. Then, the virus pellet was resuspended in PBS.

### Test solutions.

AEW solutions with various pH values were generated by the electrolysis of NaCl solution, HCl solution, or a mixture of NaCl and HCl solutions. FAC concentrations of 34.5 ± 1.9 or 23.8 ± 1.6 ppm in six different AEW solutions, named NaCl (pH 2.8 ± 0.1), NaCl (pH 4.1 ± 0.1), HCl (pH 5.4 ± 0.1), NaCl+HCl (pH 4.9 ± 0.1), HCl (pH 6.5 ± 0.2), and NaCl+HCl (pH 6.4 ± 0.1), were tested. NaCl AEW solutions at pH values of 2.8 ± 0.1 and 4.1 ± 0.1 were generated from NaCl solution using ROX-15WC (Hoshizaki Co., Ltd., Toyoake, Japan). HCl AEW solutions at pH values of 5.4 ± 0.1 and 6.5 ± 0.2 were generated from HCl solution using PURESTER μ-Clean II (Morinaga Milk Industry Co., Ltd., Tokyo, Japan). NaCl+HCl AEW solutions at pH values of 4.9 ± 0.1 and 6.4 ± 0.1 were generated from a mixture of NaCl and HCl solutions using VOX-40TA (Hoshizaki Co., Ltd.). After the electrolysis, the FAC concentration and pH of each AEW solution were adjusted by adding ultrapure water (UPW), HCl, or alkaline solution. The molar concentrations of NaCl and HCl in each AEW were unknown. NaCl AEW (pH 3.1 ± 0.0; FAC, 0.0 ppm) was prepared by placing NaCl AEW (pH 2.8 ± 0.1; FAC, 34.5 ppm) at 25°C for more than 1 week, uncovered and without shading. NaCl AEW solutions at pH values of 5.5 ± 0.1 and 6.4 ± 0.0 (FAC, 0.0 ppm) were prepared by adding NaOH solution to NaCl AEW (pH 3.1 ± 0.0; FAC, 0.0 ppm). UPW was used as the control solution. In experiments in which the virus solution and AEW were mixed, the pH and FAC concentration in the AEW were measured using a compact pH meter (Horiba Co., Ltd., Kyoto, Japan) and an AQUAB AQ-202 chlorine meter (Sibata Scientific Technology Ltd., Tokyo, Japan) just before mixing it with the virus solution.

### Evaluation of the virucidal activity of the test solutions.

SARS-CoV-2-containing VGM with 1%, 20%, or 40% FBS (viral titer, 4.9 or 6.9 log_10_ TCID_50_/mL), BCoV-containing VGM with 1% FBS, or BCoV-containing PBS (viral titer, 4.3 log_10_ TCID_50_/mL) were mixed with each test solution (UPW or each AEW solution with an FAC concentration of 0.0, 23.8 ± 1.6, or 34.5 ± 1.9 ppm) at a ratio of 1:9, 1:19, or 1:49 by volume. After 20 s, 3 h, or 24 h at 25°C, the mixture was inoculated into cells cultured in VGM with 10 mM sodium thiosulfate, which is a neutralizer of chlorine. After 10-fold serial dilution of the mixture, the virus-inoculated Vero E6/TMPRSS2 cells and HRT-18 Aichi cells were incubated for 3 and 4 days, respectively, at 37°C. The viral titer (log_10_ TCID_50_/mL) of each mixture was calculated using the Behrens-Kärber method (33). The reduction in viral titer by each AEW treatment was calculated as follows: (viral titer in UPW group) – (viral titer in each AEW group).

### Evaluation of change in pH and FAC concentration in AEW after mixing with VGM.

To measure the pH, virus-free VGM with 1% FBS was mixed with each AEW solution (FAC, 30.0 ppm) at ratios of 1:49, 1:19, and 1:9. The mixtures were incubated at 25°C for 5 min, and the pH of each mixture was measured using a portable pH meter (D-54SE; Horiba Co., Ltd.). The pH values of AEW that was not mixed with VGM (mixing ratio of VGM and AEW, 0:1) and VGM that was not mixed with AEW (mixing ratio of VGM and AEW, 1:0) were also measured. To measure the FAC concentration, virus-free VGM with 1% FBS was mixed with each AEW solution (FAC, 30.0 ppm) at a ratio of 1:9. The mixtures were incubated at 25°C for 1 min, and the FAC concentration of each mixture was measured (AQUAB AQ-201; Sibata Scientific Technology Ltd.). The FAC concentration of AEW that was not mixed with VGM (mixing ratio of VGM and AEW, 0:1) was also measured.

### Statistical analysis.

Student’s *t* tests were used to analyze the differences in the viral titers between the UPW and each AEW group. A *P* value of < 0.05 was considered to indicate a statistically significant difference.
