# Supplementary material for: Virucidal Activities of Acidic Electrolyzed Water Solutions with Different pH Values against Multiple Strains of SARS-CoV-2
Source: Appl Environ Microbiol. 2022 Dec 13;89(1):e01699-22. doi: 10.1128/aem.01699-22 (PMC9888296; doi:10.1128/aem.01699-22)
Supplement: Supplemental file 1 — Supplemental material. Download aem.01699-22-s0001.pdf, PDF file, 0.5 MB [file aem.01699-22-s0001.pdf]

Supplementary Materials for "Differences in virucidal activities of acidic electrolyzed water with different pH against multiple strains of SARS-CoV-2"

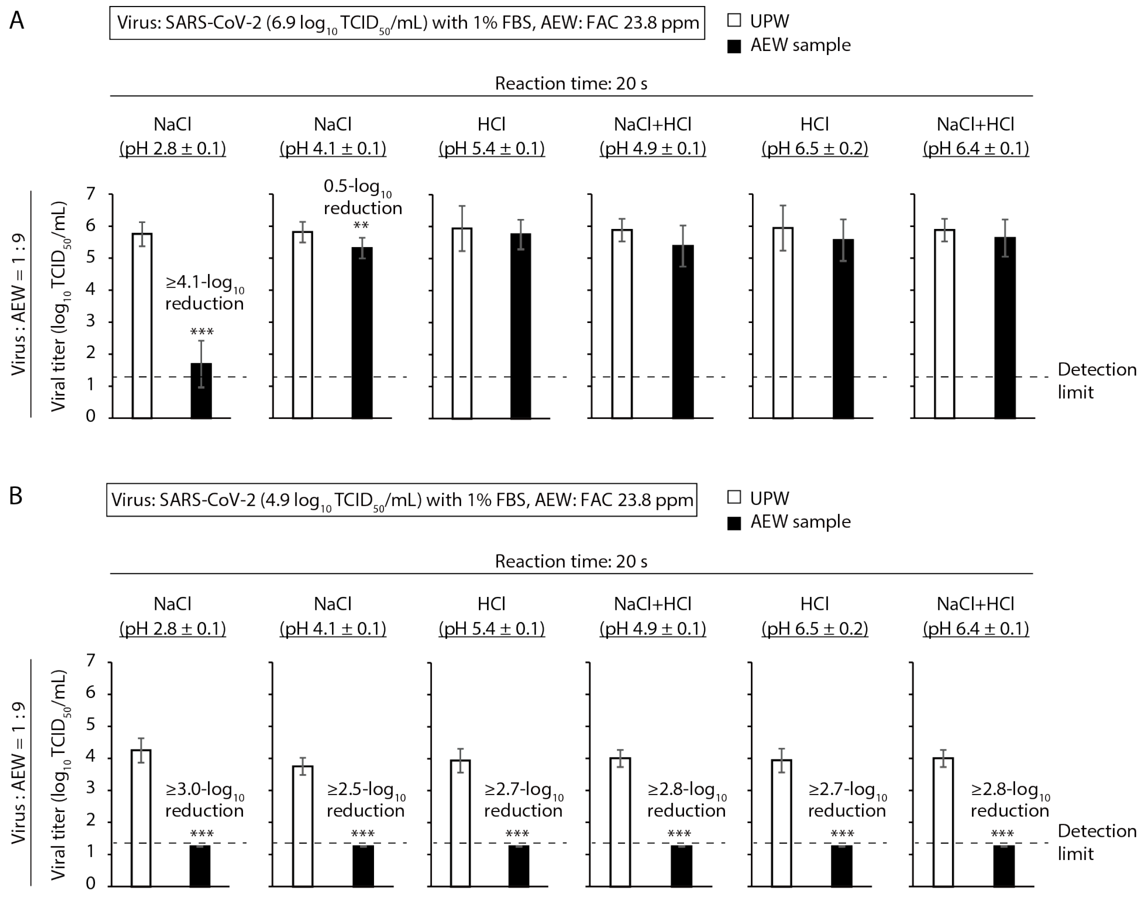

Fig. S1. Virucidal activities of various types of AEWs with low FAC concentrations against the SARS-CoV-2 ancestral stain. (A, B) SARS-CoV-2 (ancestral strain)-containing VGM with 1% FBS [viral titer: 6.9 (A) or 4.9 (B) log_{10} $TCID_{50}$/mL] was mixed with multiple AEWs (FAC concentration: 23.8 ± 1.6 ppm) at a ratio of 1:9. As a control,

virus solution was mixed with UPW. After 20 s, the viral titer of each mixture was evaluated. The detection limit of the viral titer was 1.25 $\log_{10}$ $TCID_{50}$/mL. Error bars indicate mean ± SD (n = 8). Student's t-tests were used to analyze the statistical significance of differences between UPW and each AEW group; $^{**}p < 0.01$, $^{***}p < 0.001$.

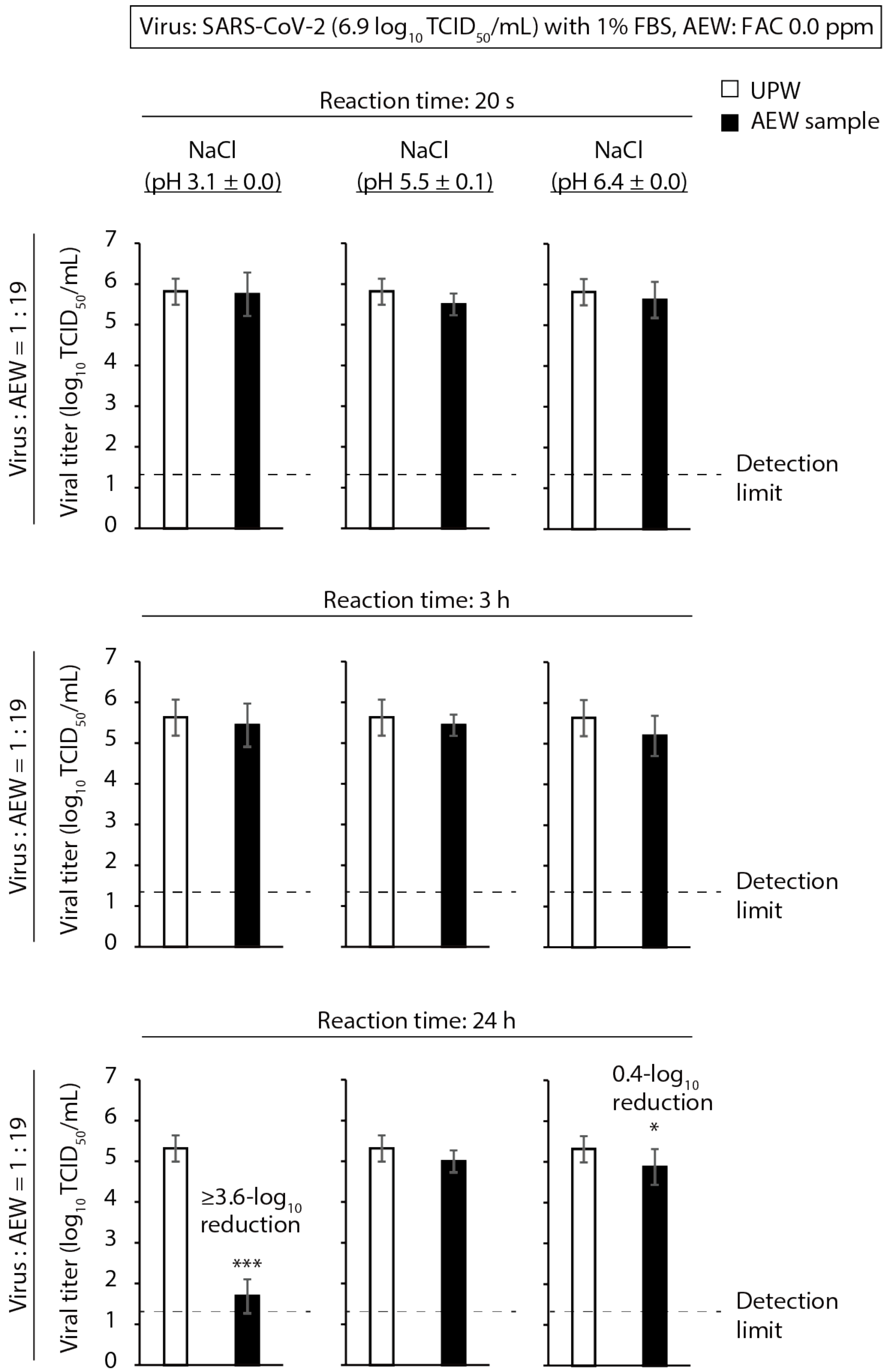

Fig. S2. Virucidal activities of FAC-free AEWs against the SARS-CoV-2 ancestral strain.

SARS-CoV-2 (ancestral strain)-containing VGM with 1% FBS (viral titer: 6.9 log_{10}

$TCID_{50}$/mL) was mixed with AEWs (FAC concentration: 0.0 ppm) with different pH at a ratio of 1:19. As a control, virus solution was mixed with UPW. After 20 s, 3 h, or 24 h, the viral titer of each mixture was evaluated. The detection limit of the viral titer was 1.25 $\log_{10}$ $TCID_{50}$/mL. Error bar indicates mean ± SD (n = 8). Student's t-tests were used to analyze the statistical significance of differences between UPW and each AEW group; **$p < 0.05$, ***$p < 0.001$.
